# Analysis of intestinal flora in elderly Uygur patients with sarcopenia

**DOI:** 10.1002/iid3.1097

**Published:** 2024-01-22

**Authors:** Zimei Shan, Na Cheng, Jia Zhu, Fei Chen, Jiani Ji, Meilibana

**Affiliations:** ^1^ People's Hospital of Xinjiang Uygur Autonomous Region Urumqi China

**Keywords:** elderly, inflammatory factors, intestinal flora, Sarcopenia

## Abstract

**Objective:**

This study was designed to analyze the structural characteristics of the intestinal flora of elderly Uygur patients with sarcopenia, thereby providing new ideas for clinical treatment.

**Methods:**

Firstly, fecal samples were collected from 40 elderly Uygur patients with sarcopenia (Sarcopenia group) and 40 healthy people (Control group). Next, significant differences in the intestinal flora between the two groups were analyzed based on 16S rDNA high‐throughput sequencing. The linear discriminant analysis effect size (LEfSe) was used to estimate the magnitude of the effect of each component (species) abundance on the differential effect. Additionally, an analysis was also performed on the relationship between the intestinal flora and the cytokines in the peripheral blood of patients with sarcopenia.

**Results:**

The results of β diversity showed that there were differences in the structure of the intestinal flora between the two groups. Besides, the phylum level of intestinal flora between the two groups was not significantly different. However, the difference was significant in the intestinal flora at the order, family, and genus levels between the two groups. Among them, *Lachnoclostridium, Photobacterium, Anaerobic Bacillus, Hydrogenophilus*, and *Eubacterium* were enriched in the Sarcopenia group; *Prevotella 9, Firmicutes FCS020 group, Streptobacillus, Aggregatibacter, Corynebacterium, Clostridium Difficile*, and *Haloanaerobium* were enriched in the Control group. The LEfSe outcomes further showed that *Lachnoclostridium* was highly enriched in the Sarcopenia group; *Prevotella 9* and *Firmicutes FCS020* group were significantly enriched in the Control group. Furthermore, the relative abundance of *Lachnoclostridium* and *Streptobacillus* were significantly different in patients with high and low IL‐6 levels.

**Conclusion:**

In conclusion, Lachnoclostridium is significantly enriched in the intestines of elderly Uygur patients with sarcopenia; the increase in Lachnoclostridium abundance and the decrease in Streptobacillus abundance are associated with high levels of IL‐6. Therefore, abnormal intestinal flora is related to inflammatory reflexes in patients with sarcopenia.

## Graphical abstract

To analyze the structural characteristics of the intestinal flora of elderly Uyghur patients with Sarcopenia. There are significant differences in the composition of intestinal flora between elderly Uyghur patients with Sarcopenia and normal individuals

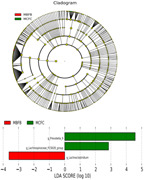

## 1 INTRODUCTION

Sarcopenia is a progressive loss of body muscle mass and/or muscle strength or muscle physiological function associated with aging, which can lead to serious negative health outcomes and disability in the elderly.^1^ As a complex disease affected by environmental and genetic factors together, the occurrence of sarcopenia involves a variety of risk factors and mechanisms.^2^, ^3^ The prevalence of sarcopenia in elderly community residents and long‐term care populations is as high as 29% and 33%, respectively. Old age, low body mass index (BMI), and low physical activity are significant risk factors leading to sarcopenia.^4^ Studies have shown that the average survival time of middle‐aged and elderly people with weaker grip is shorter than those with stronger grip.^5^ This finding indicates the important impact of sarcopenia on the elderly. In recent years, many scholars have confirmed that changes in sex‐specific hormone levels, inflammatory pathways, muscle cell apoptosis, caloric and protein intake, etc., are all related to sarcopenia.^6^ However, the specific pathogenic mechanism of sarcopenia remains to be investigated in depth. At present, resistance training and nutritional supplementation are the main methods to treat sarcopenia, but their effects are lacking.^4^ Therefore, finding new therapeutic targets is necessary to facilitate the effective treatment of sarcopenia. There are some differences in the response of ethnic minorities and Han ethnic people to sarcopenia. However, the studies on sarcopenia of ethnic minorities are rare.^7^, ^8^, ^9^, ^10^, ^11^, ^12^ Hence, it is urgent to improve the relevant research on sarcopenia of ethnic minorities.

Microbial metabolites from the intestines have been demonstrated to act as nutrients or metabolic modulators in skeletal muscles.^13^ The intestinal flora is the total set of microorganisms that colonize the human intestine, which contains about 10 trillion bacteria, and the total number of genes in the flora is 150 times the total number of genes in human cells.^14^ The intestinal flora can regulate multiple processes in the body, such as nutrient absorption, inflammation, oxidative stress, and anabolism.^15^ The composition of the intestinal flora has been confirmed to change with age from birth.^16^ Changes in intestinal microbes are related to the physiological decline of skeletal muscle function. Studies have revealed the changes in the intestinal flora of patients with cirrhosis and muscle atrophy,^17^ indicating the participation of intestinal flora in muscle atrophy. Currently, it is also reported that changes in the composition of the intestinal flora in the rat model are not only related to the decline of skeletal muscle function associated with sarcopenia but also possibly related to the level of inflammatory factors.^18^ Butyrate, the most important energy source for intestinal epithelial cells, has been reported to have a significant effect on skeletal muscles. Specifically, butyrate can help prevent skeletal muscle mass loss and maintain skeletal muscle mass via inhibiting inflammatory response and activating regulatory pathways, thereby making an increase in adenosine triphosphate (ATP) and an inhibition in muscle protein catabolism and apoptosis.^19^ However, there are few clinical studies on the relationship between intestinal flora and sarcopenia. In this study, the Uygur elderly with sarcopenia and healthy elderly were taken as research objects. Based on 16S rDNA sequencing, the difference in the abundance and diversity of the intestinal flora between the two groups was analyzed. Finally, this study revealed the internal connection between the intestinal flora and sarcopenia, explored the relationship between the intestinal flora and peripheral inflammatory cytokines tumor necrosis factor‐alpha (TNF‐ɑ), interleukin (IL)‐6, and IL‐8, and provided new ideas for the clinical treatment of sarcopenia.

## 2 MATERIALS AND METHODS

### 2.1 Clinical data

A total of 40 sarcopenia patients and 40 healthy individuals hospitalized in the People's Hospital of Xinjiang Autonomous Region from December 2018 to October 2020 were collected.

Inclusion criteria: (1) patients were aged ≥65 years and met the criteria of “decreased pace” and “decreased grip strength” in sarcopenia diagnosis; (2) people were in stable health and able to move independently; (3) the Asian Sarcopenia Working Group (AGWS) diagnostic criteria for muscle mass reduction cut‐point was considered as the diagnostic criteria of sarcopenia; (4) the appendicular skeletal muscle mass (ASM) was measured based on DM. ASM was defined as the sum of the skeletal muscle content of the upper limbs and lower limbs. ASM (kg) was divided by the square of W height (m^2^), then the skeletal muscle mass index (SMI) could be obtained. Male ≤7.0 kg/m^2^, female ≤5.4 kg/m^2^.

Exclusion criteria: patients (1) unable to move and stand up independently from the chair; (2) with physician‐diagnosed gastrointestinal disease, such as ulcerative colitis, Crohn's disease, peptic ulcer disease, gastroparesis, and so on; (3) with neurological diseases or bone and joint diseases affecting activities; (4) with chronic cardiopulmonary insufficiency (inability to carry out normal daily activities, heart failure grade III, IV, or failed to tolerate the 6 m walking test); (5) with severe renal insufficiency and needing to limit protein intake; (6) with malignant diseases, impaired cognitive function and poor compliance; (7) without the intake of antibiotics, microecological regulators and other related drugs and foods that affected the intestinal flora.

The gender, age, height, and body mass of all candidates were recorded separately, and the BMI was calculated. This research protocol was approved by the Ethics Committee of the People's Hospital of Xinjiang Uygur Autonomous Region (KY2018011842). Besides, all involved subjects were voluntary and signed an informed consent form.

### 2.2 Detection of serum biochemical indicators

The patients were fasted for 10–12 h, and the venous blood was drawn in the morning of the next day. After standing the blood samples for 1.5 h, the serum was collected by centrifugation. Then, the levels of hemoglobin (Hb), C reactive protein (CRP), total cholesterol (TC) and triglyceride (TG) in serum were detected according to corresponding biochemical kits (Nanjing Jiancheng).

### 2.3 Enzyme‐linked immunosorbent assays (ELISA)

Fasting venous blood (5 mL) of all patients was collected and stored in tubes containing ethylenediaminetetraacetic acid (EDTA). The serum was then collected after centrifugation of blood samples at 2000 g for 15 min. Subsequently, the serum levels of TNF‐ɑ, IL‐6, and IL‐8 were measured according to the instructions of the ELISA kits (Multi Sciences).

### 2.4 Extraction of bacterial genomic DNA from feces

Postfasting stool samples (3–5 g) were collected from patients using a dedicated stool specimen kit. Then, the total DNA was extracted with reference to the instructions of the stool sample DNA extraction kit (QIAGEN) and stored in the refrigerator at −80°C.

### 2.5 PCR amplification and illumina PE250 library construction

Barcode‐specific primers 515F and 907R for the 16S V3‐V4 region and the genomic DNA were used as a template. PCR was performed according to the instructions of Tks Gflex DNA Polymerase (Takara), with three replicate wells for each sample. After mixing the PCR products of the same sample, 2% agarose gel electrophoresis was performed. Next, the PCR products were recovered through cutting the gel using the AxyPrepDNA Gel Recovery Kit (AXYGEN), eluted with Tris_HCl, and then detected by 2% agarose electrophoresis. With reference to the preliminary quantitative results of electrophoresis, the PCR products were detected and quantified with QuantiFluor™ ‐ST Blue Fluorescence Quantitative System (Promega). Then, the PCR products were mixed in the corresponding proportions according to the sequencing volume requirements of each sample. Subsequently, the “Y”‐shaped adapter was connected; the self‐linked fragments of the adapter were screened and removed using magnetic beads. Finally, PCR amplification was used to enrich the library template and construct an Illumina PE250 library.

### 2.6 Bioinformatics analysis

Sequences were separated from fastq files according to the specific barcodes of each sample introduced in the Illumina PE250 library in the PCR step and spliced according to the overlap relationship. Simultaneously, the sequence quality was controlled and filtered. After distinguishing the samples, OTU cluster analysis and species taxonomy analysis were performed. Referring to the results of OTU cluster analysis, a variety of diversity index analyses were carried out, followed by the detection of sequencing depth. Additionally, a statistical analysis was performed on the community structure at each classification level based on taxonomic information.

### 2.7 Statistical analysis

Through the Qiime platform (RDP Classifier: version 2.2, confidence threshold is 0.7), α diversity and β diversity were obtained. Besides, the R language tools were employed for making graphs; SPSS26.0 software for statistical analysis; and *T*‐test for the comparison between two groups and the comparison of the median of intestinal flora. Measurement data were expressed as mean ± standard deviation (SD). The TNF‐ɑ, IL‐6, and IL‐8 levels of 40 sarcopenia patients were stratified into quartiles by the binning method, and the differences between the five most abundant bacteria at the genus level and inflammatory factors were compared; in another word, the differences of the intestinal flora between the lowest (first quartile) and the highest (fourth quartile) levels of individual cytokines were compared. The Pearson coefficient was used to analyze the correlation between inflammatory factors and intestinal flora. *p* < .05 indicated that the difference was statistically significant.

## 3 RESULTS

### 3.1 General information

There were 40 Uygur elderly with sarcopenia in the Sarcopenia group and 40 healthy Uygur elderly in the Control group. The ratio of males to females in the two groups was 25/15. There is no significant difference in age, BMI, Hb, and CRP between the two groups; the TC level of sarcopenia patients was significantly lower than that of healthy individuals. The TG level in the Sarcopenia group was significantly increased compared with that in the Control group (Table 1), indicating that the two groups were comparable.

**Table 1:** General information of subjects.

Group	Age	BMI (kg/m^2^)	Hb (g/L)	CRP (mg/L)	TC (mmol/L)	TG (mmol/L)
Sarcopenia (*n* = 40)	76.52 ± 7.40	26.49 ± 3.42	134.17 ± 13.8	4.32 ± 6.15	3.84 ± 0.89	1.45 ± 0.72
Control (*n* = 40)	74.23 ± 7.65	26.61 ± 3.96	139.76 ± 14.5	3.52 ± 4.67	4.36 ± 1.33	1.17 ± 0.46
*T*‐value	1.355	−0.140	−1.752	0.645	−2.039	2.044
*p*‐Value	.179	.889	.084	.521	.045	.044

*Note*: The value was expressed as mean ± SD.

Abbreviations: BMI, body mass index; CRP, C reactive protein; Hb, hemoglobin; TC, cholesterol; TG, triglycerides.

### 3.2 Comparison of the diversity of intestinal flora

**Figure 1:**
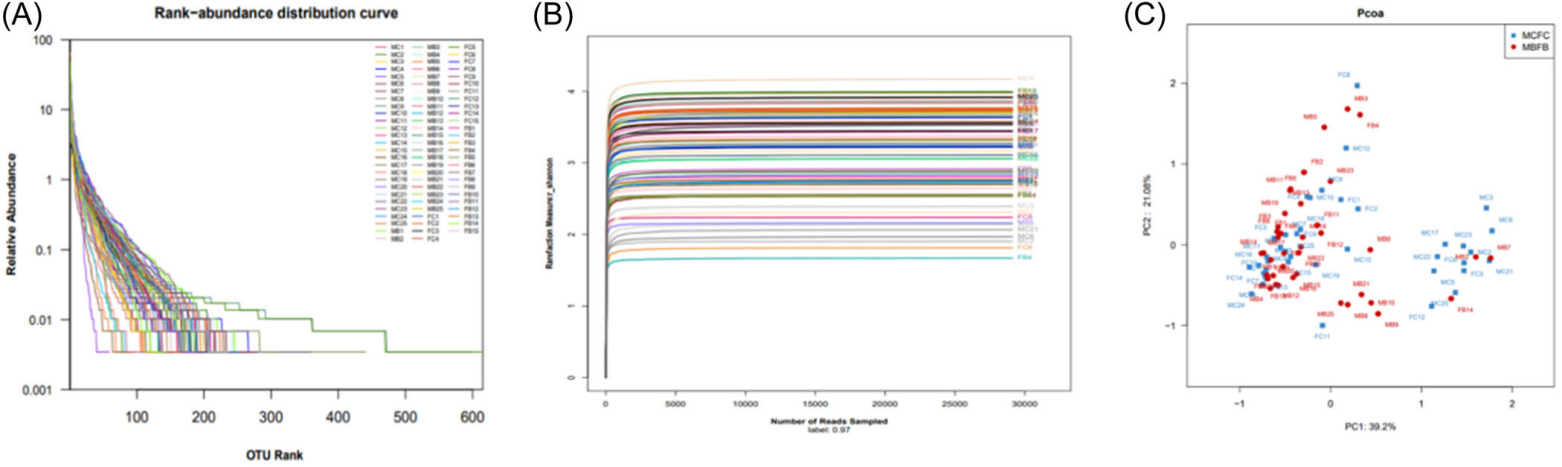
Comparison of the diversity of intestinal flora between the two groups of patients. (A) Shannon–Wiener index reflected the microbial diversity of the samples; (B) rank‐abundance curve reflected the species abundance and species uniformity of the samples from two groups of patients; (C) principal coordinate analysis (PCoA) analysis for the differences in the structure of the intestinal flora between the two groups. MBFB was the Sarcopenia group, and MCFC was the Control group.

With an average of 29,110 sequences in each stool sample, the Good's coverage index was used to analyze the sequencing coverage. The sequencing coverage of both groups reached 97%. Furthermore, the Shannon‐Wiener graph reflected the flat curve of all samples of the two sets of samples, indicating that there was enough sequencing depth for data comparison (Figure 1A). After analysis, there was no significant difference in the α diversity analysis index, Chao1 index, Ace index, Coverage index, Simpson index, and Shannon index between the two groups (Table 2). The Rank‐abundance curve further revealed the species abundance and species uniformity, indicating that the two sets of samples had smooth curves and uniform species distribution (Figure 1B). Therefore, the sample sequencing results were reliable. The principal coordinate analysis (PCoA) was further used to detect the β diversity of intestinal flora in the samples of the two groups. The results showed that the two principal coordinates contributed 39.2% and 21.08% of the difference in the intestinal flora, respectively. Moreover, the UniFrac distance between the two groups of patients was relatively long, indicating significant differences in the structure of the intestinal flora between the two groups (Figure 1C).

**Table 2:** Comparison of the diversity alpha correlation index between the two groups of patient samples.

Group	Ace index	Chao1 index	Coverage index	Shannon index	Simpson index
Sarcopenia (*n* = 40)	265.42 ± 115.24	256.3 ± 98.04	0.99 ± 0.001	3.25 ± 0.61	0.11 ± 0.09
Control (*n* = 40)	274 ± 124.69	267.63 ± 114.83	1.00 ± 0.001	3.20 ± 0.61	0.12 ± 0.10
*T*‐value	−0.319	−0.474	0.163	0.358	−0.720
*p*‐Value	.75	.637	.871	.721	.478

### 3.3 Intestinal microecological composition and distribution abundance

Based on the phylum level classification, all sample sequences belonged to 24 phylum. After detection and analysis, we found that five types of bacteria with higher abundance included *Firmicutes*, *Bacteroidetes*, *Acidobacteria*, *Proteobacteria*, and *Tenericutes*. However, there was no significant difference in the relative abundance of the five bacterial phyla between the two groups (*p* > .05, Table 3).

**Table 3:** Comparison of intestinal microbes between the two groups at the phylum level (M).

Group	*Firmicutes*	*Bacteroidetes*	*Acidobacteria*	*Proteobacteria*	*Tenericutes*
Sarcopenia (*n* = 40)	13562.70	12359.97	690.45	2209.72	250.53
Control (*n* = 40)	13678.22	12660.07	805.77	1628.45	187.40
*T*‐value	797.00	765.0	770.50	618.50	610.50
*p*‐Value	.98	.74	.78	.08	.06

*Note*: Values were expressed as medians using *t*‐test.

At the order level, the abundance of *Actinobacteria* in *Firmicutes Phylum* in the Sarcopenia group was significantly less than that in the Control group, while *Vibrio Parahaemolyticus* in the *Proteobacteria Phylum* was notably increased (Table 4).

**Table 4:** Comparison of intestinal microbes between the two groups at the order level (M).

Group	Number of cases	*Actinobacteria*	*Vibrio Parahaemolyticus*
Sarcopenia	40	0.45	0.5
Control	40	1.075	0.15
*T*‐value		606	662
*p*‐Value		.021	.027

*Note*: Values were expressed as medians using *t*‐test.

At the family level, *Bacillus* and *Vibrionaceae* in the Sarcopenia group increased significantly compared with those in the Control group. However, *Corynebacteriaceae* was significantly enriched in the Control group (Table 5).

**Table 5:** Comparison of intestinal microbes between the two groups at the family level (M).

Group	Number of cases	*Bacillus*	*Vibrionaceae*	*Corynebacteriaceae*
Sarcopenia	40	2.9	0.45	0.5
Control	40	0	0.15	0.875
*T*‐value		700	662	632
*p*‐Value		.022	.027	.043

*Note*: Values were expressed as medians using *t*‐test.

At the genus level, the relative abundance of *Lachnoclostridium*, *Photobacterium*, *Anaerobic Bacillus*, *Hydrogenophilus*, *Eubacterium* in the Sarcopenia group was higher than those in the Control group. *Prevotella 9*, *Firmicutes FCS020 group*, *Streptobacillus*, *Aggregatibacter*, *Corynebacterium*, *Clostridium Difficile*, and *Haloanaerobium* were more enriched in the control group than those in the Sarcopenia group (Table 6).

**Table 6:** Comparison of intestinal microbes between the two groups at the genus level (M).

Group	Sarcopenia group (*n* = 40)	Control group (*n* = 40)	*T*‐value	*p*‐Value
*Photobacterium*	0.225	0	660	.006
*Streptobacillus*	2.175	187.725	623.5	.011
*Prevotella 9*	2324.925	4514.575	559.5	.019
*Anaerobic Bacillus*	2.900	0	700	.022
*Aggregatibacter*	0.025	1.425	678.5	.025
*Hydrogenophilus*	0.675	0.325	649.5	.029
*Rhodobacteraceae_uncultured*	0.125	0	720	.043
*Eubacterium*	3.525	0	720	.043
*Subgroup 7_norank*	0	0.900	720	.043
*Corynebacterium*	0.500	0.875	632	.043
*Clostridium Difficile*	0.400	12.450	654	.045
*Lachnoclostridium*	392.125	142.25	592	.046
*Halaanaerobium*	0.950	4.475	681	.047
*Prevotella*	0.425	5.300	646	.048
*Firmicutes FCS020 group*	6.875	10.300	595	.048

*Note*: Values were expressed as medians using *t*‐test.

### 3.4 Analysis of intestinal flora composition among samples of each group

**Figure 2:**
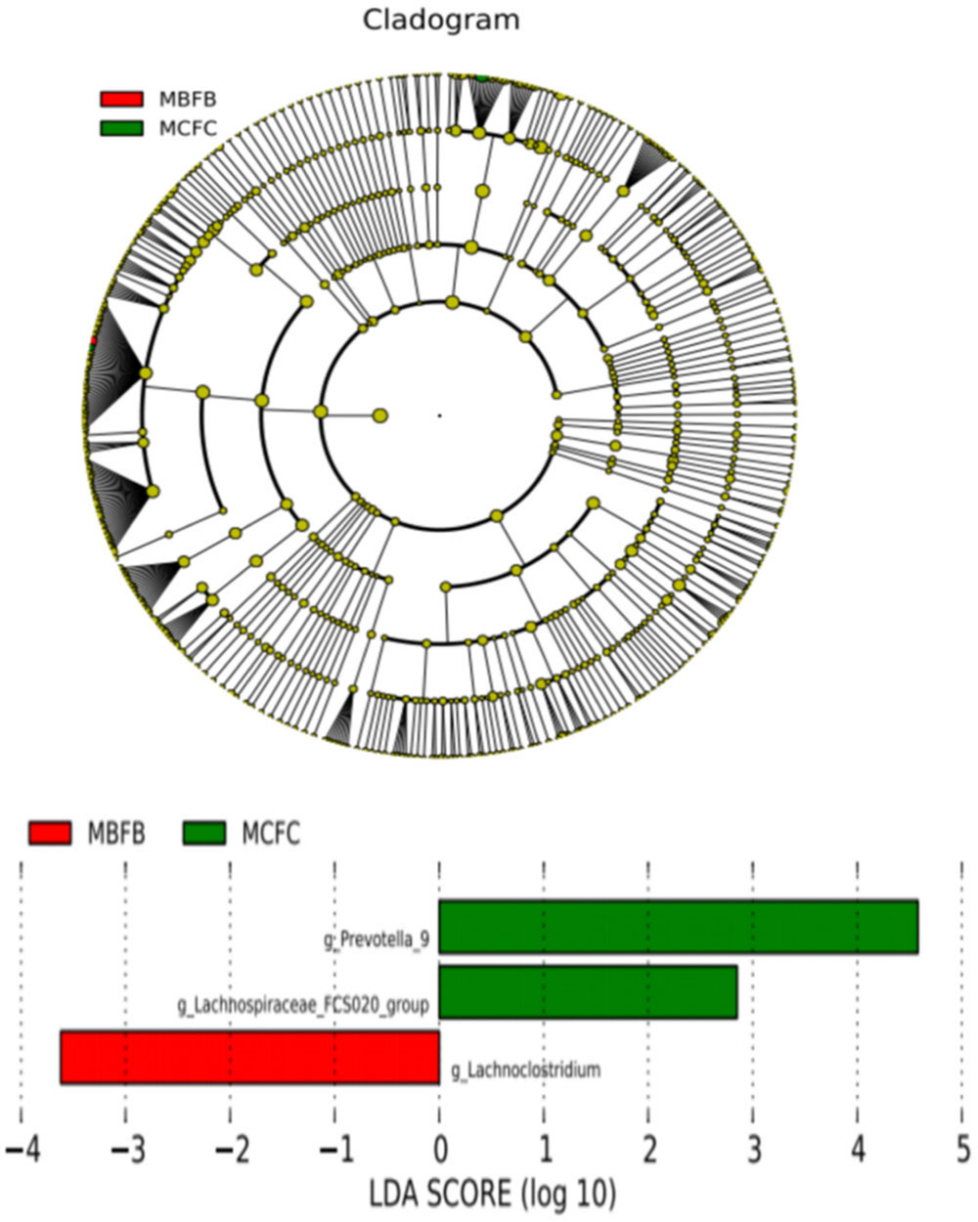
Linear discriminant analysis effect size (LEfSe) analysis for significant changes in intestinal flora types between the two groups of patients. MBFB represented the Sarcopenia group, and MCFC represented the Control group.

Furthermore, linear discriminant analysis effect size (LEfSe) was used to analyze the differences in the flora between the two groups, and the specific main flora between the two groups was searched. The analysis results showed that the relative abundance of *Lachnoclostridium* in the Sarcopenia group was higher than that in the Control group, and the difference was the most significant. *Prevotella 9* and *Firmicutes FCS020 group* were only enriched in the Control group, and the difference was most significant from the Sarcopenia group (Figure 2).

### 3.5 Correlation analysis of intestinal flora and inflammatory cytokines

The TNF‐ɑ, IL‐6, and IL‐8 levels of 40 sarcopenia patients were stratified according to the quartiles. Besides, the median and interquartile range [M (P_25_, P_75_)] were used for comparison and expressed as the difference in intestinal flora between the lowest (first quartile) and highest (fourth quartile) levels of a single cytokine and the correlation between inflammatory factors and intestinal flora. The relative abundance of *Lachnoclostridium* in patients with high levels of IL‐6 was higher than that in patients with low levels of IL‐6 (*F* = 2.27, *p* < .05). Moreover, the relative abundance of *Streptobacillus* in patients with low IL‐6 levels was higher than that in patients with high levels of IL‐6 (*F* = 2.17, *p* < .05), with a statistical significance (*r* = −0.27, *p* < .05). Overall, the relative abundance of *Streptobacillus* was negatively correlated with the level of IL‐6. However, the colony composition was not significantly different between sarcopenia patients with high and low levels of TNF‐ɑ and IL‐8 (*p* > .05) (Tables 7, 8, 9).

**Table 7:** Comparison of tumor necrosis factor‐alpha (TNF‐ɑ) and intestinal flora.

Group	*Streptobacillus*	*Prevotella 9*	*Lachnoclostridium*	*Eubacterium*	*Firmicutes FCS020*
TNF‐ɑ	0.16 (8.75, 0.20)	1.29 (1, 0.22)	159 (234.5, 145)	17 (17.6, 42.5)	2.22 (3, 2.57)
*T*‐value	0.98	1.06	1.29	−0.62	0.14
*F*‐value	1.19	1.12	0.33	0.29	0.13
*R*‐value	−0.11	−0.15	−0.14	0.08	−0.01

*Note*: Values were given in M (P25, P75).

**Table 8:** Comparison of interleukin (IL)‐6 and intestinal flora.

Group	*Streptobacillus*	*Prevotella 9*	*Lachnoclostridium*	*Eubacterium*	*Firmicutes FCS020*
IL‐6	0.16 (1, 0)	1.23 (904, 0.8)	159 (85, 156)	17 (50, 33)	2.5 (0.67, 3)
*T*‐value	1.26	0.80	−1.07	−0.22	−0.07
*F*‐value	2.17*	0.49	2.27*	0.45	0.99
*R*‐value	−0.27	−0.061	−0.13	−0.04	−0.01

*Note*: Values were given in M (P25, P75). *p* < .05 indicated a statistical significance.

**Table 9:** Comparison of interleukin (IL)‐8 and intestinal flora [M (P_25_, P_75_)].

Group	*Streptobacillus*	*Prevotella 9*	*Lachnoclostridium*	*Eubacterium*	*Firmicutes FCS020*
IL‐8	0.16 (0, 5.38)	1.29 (401, 7.2)	159 (245.67, 154)	17 (1.3, 2.0)	2.22 (2.33, 3.67)
*T*‐value	−0.73	−0.12	0.19	−1.53	0.15
*F*‐value	0.53	0.27	0.17	0.66	0.17
*R*‐value	0.02	0.03	0.07	0.16	0.17

*Note*: The value was represented by M (P_25_, P_75_).

## 4 DISCUSSION

Sarcopenia is not only associated with an increased risk of falls, fractures, and disability,^20^, ^21^ but also correlated with metabolic disorders such as insulin resistance, type 2 diabetes, dyslipidemia, and hypertension.^22^ The essence of sarcopenia is the decrease in the number and cross‐sectional area of muscle fibers and the net degradation of protein, which are closely related to factors such as increased inflammation, oxidative stress damage, mitochondrial dysfunction, abnormal autophagy, and dysregulation of muscle quality regulatory factors. At present, there are few studies on the pathogenesis and mechanism of sarcopenia domestically and abroad, especially on the pathophysiological mechanism. The intestinal flora mainly includes the dominant flora composed of obligate anaerobes and the secondary flora composed of aerobes or facultative anaerobic bacteria, which can be divided into three types of flora: beneficial bacteria, harmful bacteria, and neutral bacteria. Research on the characteristics of the intestinal microecological structure reveals the changes in the composition of the intestinal flora. Specifically, with the increase of age, the composition of the intestinal flora of the elderly decreases from the number and proportion of beneficial bacteria such as bifidobacteria and lactic acid bacteria to the increase in the number and proportion of harmful bacteria such as toxin‐producing gram‐negative bacteria, and so on, accompanied by a decrease in the number of butyrate‐producing bacteria. Intestinal microdysbiosis can affect the tight junctions of intestinal epithelial cells, leading to increased intestinal wall permeability. Then, gram‐negative bacilli lipopolysaccharide and other substances can enter the circulation from the intestinal lumen, causing the release of cytokines and the body's oxidative stress response. There is a previous research that changes in intestinal flora are related to the decline of skeletal muscle function.^18^

The Shannon and Simpson indices are used to illustrate community diversity and reflect the number and abundance of species; the Ace index and Coverage index are adopted to calculate the richness of species by counting the number of species that have not been observed.^23^ In this study, the α diversity analysis index showed that Ace index, Coverage index, Simpson index, and Shannon index were not significantly different between the two groups, indicating that the species abundance was the same and the species distribution was uniform. Hence, the sample sequencing results were reliable. Subsequently, the high‐throughput 16S sequencing method was used to detect the difference in intestinal flora between sarcopenia patients and normal individuals. Briefly, in contrast to the Control group, the relative abundance of *Lachnoclostridium* in the Sarcopenia group was higher while the relative abundance of *Prevotella 9 Genus* and *Firmicutes FCS020* group was lower. Studies have shown the ability of *Lachnoclostridium* to produce short‐chain fatty acids, the most common of which is butyrate.^24^ Walsh et al.^25^ proposed that supplementation with butyric acid in 16‐month‐old mice prevented hindlimb muscle atrophy by inhibiting histone deacetylase (histone deactylase55). However, Kang et al. claimed that in the butyric acid‐producing bacteria genus, there was less *Lachnoclostridium* while more *Lacobacillus* in sarcopenia patients.^26^ This was inconsistent with the results of our article, possibly owing to the susceptibility of intestinal flora to diseases, age, eating habits, geographic location, and other factors. In humans, treatment of elderly patients with prebiotics containing FOS and inulin for 13 weeks improved muscle function, as shown by decreases in exhaustion and improved handgrip strength. Taken together, these findings indicate that muscle mass and functions are closely associated with the composition of gut microbiota.^27^ Overall, the increase in the relative abundance of Lachnoclostridium in elderly Uygur patients with sarcopenia is of great significance because it delays the progression of the disease through promoting the production of butyrate, maintaining the barrier function of intestinal epithelial cells and reducing intestinal microbiota imbalance.

Sarcopenia has been shown to be associated with chronic, low‐grade, and systemic inflammation.^28^ “Inflamm‐aging” suggests that the aging process accompanies with the presence of pro‐inflammatory factors.^29^ Cytokines IL‐6, IL‐8, TNF‐α, and cell chemokines are all independently related to aging. Inflammation is correlated with the catabolism of skeletal muscle and fat and can cause anorexia, weight loss, and sarcopenia.^30^, ^31^ Higher levels of circulating inflammatory markers (IL‐6 and TNF‐α) are significantly associated with lower skeletal muscle strength and muscle mass.^32^ Studies have suggested that different levels of serum inflammatory factors and the abundance of certain intestinal bacteria may affect the pathophysiology of debilitation of sarcopenia.^33^ In this study, the binning method was used to stratify the levels of TNF‐ɑ, IL‐6, and IL‐8 in 40 sarcopenia patients in quartiles. Briefly, the relative abundance of *Lachnoclostridium* in patients with high IL‐6 levels was increased compared with that in patients with low IL‐6 levels. Besides, patients with low IL‐6 level had a higher relative abundance of *Streptobacillus* than those with high IL‐6 level, which is negatively correlated; while TNF‐ɑ and IL‐8 were not significantly correlated with their differential colonies. IL‐6 is a complicating factor in the causality of sarcopenia because it causes insulin resistance in older adults.^34^, ^35^, ^36^ Thus, the reduced relative abundance of streptococcus in patients with high levels of IL‐6 may be related to interspecific competition, in which the relative abundance of Lachnoclostridium in the gut increases, suppressing the number of streptococcus. But this is just our hypothesis, and more data are required to support such hypothesis.

In this study, there are still many issues that need to be further addressed. For instance, with the continuous development of microbiological and metabolomic techniques, the relationship between dysbiosis of the gut flora and sarcopenia in the elderly continues to be explored, but the exact mechanisms remain unclear. Dysbiosis of the gut flora has a high correlation with sarcopenia, which leads to reduced physical function or even disability, and consequently affects the standard of living and ability to live on one's own in the elderly. Targeted modulation of dysbiosis may provide new ideas for treatment. In addition, butyrate plays an inprotant role in preventing loss of skeletal muscle mass, maintaining skeletal muscle mass and protecting intestinal flora. Therefore, it is extremely important to investigate the changes in the content of butyrate in elderly Uygur patients with sarcopenia.

## 5 CONCLUSION

In summary, there are significant differences in the composition of intestinal flora between elderly Uygur patients with sarcopenia and normal individuals. *Lachnoclostridium* and *Eubacterium* are significantly enriched in sarcopenia patients, while *Streptobacillus*, *Firmicutes FCS020* group, and *Prevotella 9* are significantly reduced. The high level of IL‐6 in patients is related to the increase of *Lachnoclostridium* abundance and the decrease of *Streptobacillus* abundance, indicating that the abnormal intestinal flora is related to the inflammatory response in sarcopenia patients. All in all, targeting intestinal dysbacteriosis may be an effective direction for the treatment of sarcopenia.

## AUTHOR CONTRIBUTIONS

All authors contributed to the study conception and design. Material preparation, data collection and analysis were performed by Zimei Shan, Na Cheng, and Jia Zhu. The first draft of the manuscript was written by Fei Chen, Jiani Ji, Meilibana and all authors commented on previous versions of the manuscript. All authors read and approved the final manuscript.

## CONFLICT OF INTEREST STATEMENT

The authors declare no conflict of interest.

## Data Availability

The data used to support the findings of this study are available from the corresponding author upon request.
